# Activating the Hippo pathway by nevadensin overcomes Yap-drived resistance to sorafenib in hepatocellular carcinoma

**DOI:** 10.1007/s12672-023-00699-y

**Published:** 2023-05-27

**Authors:** Hewen Shi, Ying Zou, Xiaoxue Wang, Guoli Wang, Yijia Gao, Fan Yi, junqing Xu, Yancun Yin, Defang Li, Minjing Li

**Affiliations:** 1 Featured Laboratory for Biosynthesis and Target Discovery of Active Components of Traditional Chinese Medicine, School of Integrated Traditional Chinese and Western Medicine, Binzhou Medical University, Yantai, Shandong People’s Republic of China; 2 School of Basic Medical Sciences, Binzhou Medical University, Yantai, Shandong People’s Republic of China; 3 Department of Hematology, Qingdao University Medical College, Affiliated Yantai Yuhuangding Hoepital, Yantai, Shandong People’s Republic of China

**Keywords:** Hippo pathway, Nevadensin, YAP, Sensitization

## Abstract

**Background:**

Hepatocellular carcinoma (HCC) is a highly malignant type of tumor that is insensitive to cytotoxic chemotherapy and often develops drug resistance. Nevadensin, a bioflavonoid, exhibits anti-cancer properties in some cancers. However, the precise underlying mechanism of nevadensin against liver cancer are poorly understood. We aim to evaluate the efficacy as well as the molecular mechanism of nevadensin in the treatment of liver cancer.

**Methods:**

Effects of nevadensin on HCC cell proliferation and apoptosis were detected using EdU labeling and flow cytometry assays. The molecular mechanism of nevadensin on HCC was determined using RNAseq. The effects of nevadensin on hippo-Yap signaling were verified using western blot and RT-PCR.

**Results:**

In this study, we show that nevadensin significantly inhibits growth of HCC cells via inducing cell cycle arrest and apoptosis. RNAseq analysis showed that nevadensin regulates multiple functional signaling pathways associated with cancer including Hippo signaling. Western Blot analysis revealed that nevadensin notably induces activation of the MST1/2- LATS1/2 kinase in HCC cells, further resulting in the primary effector molecule YAP phosphorylation and subsequent degradation. These results indicated that nevadensin might exert its anti-HCC activity through the Hippo-ON mechanism. Moreover, nevadensin could increase the sensitivity of HCC cells to sorafenib by down-regulating YAP and its downstream targets.

**Conclusions:**

The present study indicates that nevadensin could be a potential effective approach to treating HCC, and overcoming sorafeni resistance via inducing activation of Hippo signaling.

## Introduction

Liver cancer is the second most prevalent type of cancer, which leads to > 8,00,000 deaths each year. It is regarded as a global health problem with the increase in morbidity and mortality [1]. The most common type of liver cancer, known as hepatocellular carcinoma (HCC), accounts for more than 90% of primary liver cancers [1–3]. Currently, according to the Barcelona Clinic Liver Cancer (BCLC) staging system, the effective therapies treat the different stages of HCC including hepatic resection, liver transplantation, tumor ablation, chemoembolization, and systemic therapy [4]. In recent years, radiofrequency ablation combined with transarterial chemoembolization or arterial embolization therapy have been identified as the first-line treatment for patients with early HCC [5, 6]. However, most patients with HCC are diagnosed at late stages, when the beneficial treatments of hepatic resection, ablation, and liver transplantation cannot be applied. Although many people have tried to develop molecular targeted drugs and immunotherapeutic methods, no effective drugs have been developed for the treatment of liver cancer. Furthermore, existing drugs are reported to cause a wide range of multi-system adverse reactions [7]. Therefore, there is a need to develop new treatment options for HCC patients.

Recently, the screening of anticancer compounds from natural plant components is one of the important research methods for medical scholars at home and abroad. Flavonoids are secondary metabolites produced by plants during long-term natural selection and widely exist in People’s Daily diet, such as fruits, vegetables, a variety of dietary supplements and Chinese herbal medicine. Previous studies have shown that non-toxic flavonoids, mainly exist in natural plants, are of great significance for the prevention and treatment of human tumors, aging, cardiovascular diseases and other degenerative diseases [8, 9].

Nevadensin (5, 7-Dihydroxy-4’, 6, 8, - methoxyflavone), also known as Lysionotin (Lys), is described as a flavonoid compound that is widely distributed in *Lysionotus pauciflora* maxim. It exhibits many in vitro pharmacological and biological activities such as anti-inflammatory, anti-microbial, and anti-tuberculosis [10, 11]. In addition, studies have shown that nevadensin induced the intrinsic apoptosis pathway by interrupting the cell cycle and activating caspase-3 and caspase-9, leading to DNA damage and apoptosis in human colon cancer HT29 cells [12]. It was also associated with caspase-3-mediated mitochondrial apoptosis in many HCC cells, such as BEL-7404, HepG2, and SMMC-7721 [13, 14]. Compared to colon cancer-based HT29 cells, HCC HepG2 cells were more sensitive to nevadensin treatment [15]. However, more research is needed to understand the mechanisms used by nevadensin for treating HCC.

In this study, RNAseq analysis was conducted to investigate the molecular mechanism via which nevadensin inhibits the proliferation of HCC. The Ballgown software was employed for statistically analyzing the mRNA expression levels derived by the whole transcriptome sequencing. KEGG enrichment analysis revealed that differentially expressed genes were enriched in signaling pathways treated by nevadensin including the Hippo pathway, cell cycle, cell apoptosis, and others.

The hippo pathway is an evolutionarily-conserved signaling pathway, which is a major regulator of liver growth, size, development, metabolism, regeneration, and homeostasis [16, 17]. It is a classical kinase cascade that phosphorylates the Mst1/2-sav1 complex and activates the phosphorylation of the LATS1/2-mob1A/B complex for inactivating Yap and TAZ [18, 19]. These kinases and scaffolds were regarded as primary regulators of the Hippo pathway, and help in activating a variety of carcinogenic processes [20]. Among them, Yap/TAZ was seen to be the main effector molecule, which was downstream of the Hippo pathway, and its abnormal activation was related to a variety of human cancers including liver cancer [18, 21]. Currently, Yap/TAZ inhibitors offered promising clinical outcomes, however, since Yap/TAZ played a variety of roles in cancer promotion and tumor regeneration, the Hippo pathway has emerged as an attractive but difficult target in recent drug development research [22–28].

In this study, we found nevadensin could induce HCC cells apoptosis and cell cycle arrest. Furthermore, we will intend to explore how nevadensin activates the hippo pathway for improving the anti-tumor effects in HCC cells. Earlier studies have indicated that high YAP expression levels are accompanied by patient resistance to sorafenib. Therefore, we will plan to ensure whether nevadensin could down-regulate the Yap expression and increase the efficacy of sorafenib.

## Materials and methods

### Reagents and antibodies

The biochemical reagent Nevadensin (#3807) was purchased from Nature Standard (Shanghai, China). MG132 (#HY-13,259) and Sorafenib (#HY-10,201) were purchased from MedChem Express. The following antibodies were Used: YAP (#14074T), phospho-YAP (#13619T), MST1(#3682T), phospho-MST1/2 (49,332 S), LATS1 (#3477T), phospho-LATS1 (#8654S), Merlin (#12888T), phospho-Merlin ( #13281S), Cleaved Caspase-3 (#9611S), Cleaved Caspase-9 (#20750S), Cleaved PARP (#5625S), CDK6 (#13,331), Anti-rabbit IgG (#7074), Anti-mouse IgG (#7076) were purchased from Cell Signaling Technology ; CDK4 (#11026-1-AP), Cylcin B1 (#28603-1-AP) were purchased from proteintech; β-actin ( #TA-09 ) was purchased from ZSGB-Bio; DNA Content Quantitation Assay (Cell Cycle) (#CA1510), Hoechst 33,258 (IH0060), CCK-8 Cell Proliferation and Cytotoxicity Assay Kit was (CA1210) purchased from Solarbio. PrimeScript™ RT reagent Kit with gDNA Eraser (Perfect Real Time) purchased from TaKaRa. FastStart Universal SYBR Green Master (ROX) (#4,710,436,001) purchased from Roche. Cell-Light EdU Apollo 488 in Vitro Kit (100T) (#C10310-1) purchased from RiboBio.

### Cell culture

The HepG2 and Hep3B cell lines were acquired from Procell Life Science & Technology Co., Ltd. (China). The cell lines were cultured using DMEM (Gibco, NY, USA) that was supplemented with 10% (v/v) Fetal Bovine Serum (FBS, Hyclone, USA), and antibiotic solution (1:1, penicillin + streptomycin, 100 U/ml). Thereafter, the cell suspension was transferred to a new cell culture bottle, and incubated in the sterile cell incubator with 95% air and 5% CO_2_-saturated humidity, at 37 °C. After 24 h, cell status and adhesion were observed, and the cells with good growth status were selected for subsequent experiments. The culture medium was replaced with a fresh medium for further culturing.

### Cell morphology

The HepG2 and Hep3B cells (in the logarithmic growth stage) were selected and washed using PBS. Then, trypsin (0.25% v/v) was added to the cell suspension for thorough digestion. The washed cell pellet was suspended in DMEM medium containing 10% calf serum, diluted to 3 × 10^4^ cells.ml^− 1^, and inoculated in 12-well culture plates. After culturing the cells for 24 h, the plates were processed according to different experimental groups. Finally, the cell morphology and growth state were observed by taking pictures with optical microscope.

### Cell viability assays

5 × 10^3^ cells were seeded in 96-well plates and then treated according to the experimental requirements. After incubation, the 96-well plate was placed on the ultra-clean workbench, the original medium was discarded, and CCK-8 working solution (100 µl) was added to every culture well and incubated for 2 h in a sterile cell incubator. A microplate reader was used to measure the A_450_ values of every well. Thereafter, the inhibition rates in the cells treated with nevadensin/ sorafenib or both were calculated. All the experiments were performed in triplicate.

### Hoechst 33258 staining assay

Cells (0.5-1 × 10^3^) were seeded into the 24-well culture plates and incubated in the presence or absence of nevadensin. The cells were treated with the Hoechst 33,258 staining solution (1:100 dilution with PBS) and used as the working solution. This working solution (1 ml) was introduced into every 24-well culture plate and incubated at 37 °C for 30 min. After 30 min, the staining solution was discarded and the cells were rinsed with PBS thrice. Then, the apoptotic cells were observed by fluorescence microscopy and the bright blue cell nuclei were stained. All the experiments were performed in triplicate.

### Flow cytometry

Flow cytometry was used for identifying cell apoptosis with the help of an Annexin V-FITC/PI kit, following the manufacturer’s instructions. The cells were treated as per the requirements, and the cells in every well were collected and centrifuged at a rate of 800 × g for 3 min. The cell-free supernatant was discarded, and the cells were rinsed with PBS buffer and centrifuged at a rate of 800 × g for 3 min.

Then, the cells were stained with Annexin-V or PI solution (5 µl), and incubated in the darkness for 15 min, at room temperature. Thereafter, the scatter plots, and the distribution of red fluorescence- (PI) and green fluorescence (FITC)-stained cells were observed using the flow cytometry process (BD Biosciences, CA, USA). The apoptosis scatter plots were plotted to analyze the cell apoptosis rate. All the experiments were performed in triplicate.

Finally, the HCC cell suspension (1 × 10^6^ cells; 100 µl) was fixed using 70% ice-cold ethanol for 1 h, respectively. After ribonuclease A treatment, the cell suspension was co-cultured with the cell punching agent and PI for 15 min, rinsed with PBS, and finally resuspended in PBS. Flow cytometry was used for detecting the cell cycle distribution as per the distribution of DNA content. All the experiments were performed in triplicate.

### EdU imaging assay

Cells (0.5–1 × 10^6^) were seeded into the 6-well culture plates and treated with or without nevadensin. Then, cells were stained with 50 µM EdU medium for 2 h. The cells, in turn, were immobilized with 4% polyoxymethylene for 30 min, treated with 2 mg/ml glycine for 5 min, permeated by 0.5% TritonX-100 for 10 min. After that, cells were treated with Apollo 567 for 30 min at room temperature avoiding light, and still permeated by 0.5% TritonX-100 for 20 min. The DNA replication activities in the S-phase of the cell cycle were determined using fluorescence microscopy, and the cell proliferation ability was detected rapidly and sensitively. All the experiments were performed in triplicate.

### RNAseq and data analysis

The Hep3B cells, treated with or without nevadensin (25µΜ) for 24 h, were collected and subjected to RNA sequencing (BGI Genomics, China). The following procedure was used for sequencing: Extracting total RNA; quality inspection; constructing a library; computer sequencing; Data filtering; quality control; Reference genome alignment; Overall quality assessment; and analyzing the final data.

For analyzing the RNAseq data, generated from nevadensin-treated Hep3B cells and GEO (Gene Expression Omnibus) datasets (GSE189711, GSE121153), the raw Fastq files were aligned with the hg19 and mm10 reference genomes, respectively.

### Western blot analysis

HCC cells were collected and lysed using a certain amount of RIPA lysate based on the cell number. After lysis, the samples were centrifuged, and the cell-free supernatant was collected. The total protein concentration in the supernatant was quantified by the BCA method. Then, the lysates were boiled in SDS polyacrylamide gel electrophoresis (SDS-PAGE) sample loading buffer for 5–10 min at 99 °C. Subsequently, the cell protein lysate (30 µg) was electrophoresed using the SDS-Polyacrylamide gel electrophoresis (SDS-PAGE) technique and transferred to the PVDF membrane. The PVDF membrane was blocked, incubated with specific primary antibodies, and then the membrane was washed. Thereafter, the membrane was incubated with a specific secondary antibody and then, the membrane was washed. Finally, the ECL chemiluminescence solution was utilized for detecting the expression and phosphorylation levels of the related proteins. All the experiments were performed in triplicate.

### Real-time PCR

The HepG2 and Hep3B cells were planted in 6-well plates and then treated according to the experimental requirements. Then, total RNA was extracted from the lysed cells using the Trizol Reagent (Invitrogen, USA), and cDNA was synthesized using random hexamers from mRNA with the aid of the Script^™^RT reagent Kit with gDNA Eraser (Perfect Real Time) (TaKaRa, JPN). Quantitative real-time PCR was conducted using LightCycler system according to the manufacturer’s instruction. The final reaction system used for PCR synthesis was as follows: RNase-free water −2.4 µl; Forward and Reverse Primers −0.3 ul, each; cDNA was diluted 10 times, and 2 µl of the diluted cDNA solution was mixed with FastStart Universal SYBR Green Master (5 µl of ROX). The PCR reaction was carried out using the following conditions: Initial denaturation at 95 °C for 5 min; 40 denaturation cycles at 95 °C for 15 s; and annealing at 60 °C for 60 s. The sequence of primers used were as follows: human GAPDH Sequence (5′to3′) F:CATGAGAAGTATGACAACAGCCT;R: AGTCCTTCCACGATACCAAAGT; human CTGF Sequence (5′to3′) F: CAGCATGGACGTTCGTCTG; R: AACCACGGTTTGGTCCTTGG: human ITGAV Sequence (5′to3′) F: ATCTGTGAGGTCGAAACAGGA; R: TGGAGCATACTCAACAGTCTTTG; human ITGB5 Sequence (5′to3′) F: TCTCGGTGTGATCTGAGGG; R: TGGCGAACCTGTAGCTGGA; human SMAD7 Sequence (5′to3′). F: TTCCTCCGCTGAAACAGGG; R: CCTCCCAGTATGCCACCAC. The GAPDH was used for normalization. All the experiments were performed in triplicate.

### Statistical analysis

The statistical analyses were conducted using SPSS 18.0 and GraphPad Prism 5.0 software. Results, derived from at least three times experiments, are expressed as mean ± Standard deviation (SD). Two tests—the post hoc multiple comparison Bonferroni tests and the one-way analysis of variance (ANOVA) test—were employed to evaluate the significant differences between the two groups. All the data are represented as mean ± SD. * p < 0.05 and ** p < 0.01 were considered statistically significant.

## Results

### Nevadensin inhibits cell viability and induces apoptosis in HCC cells

**Fig. 1:**
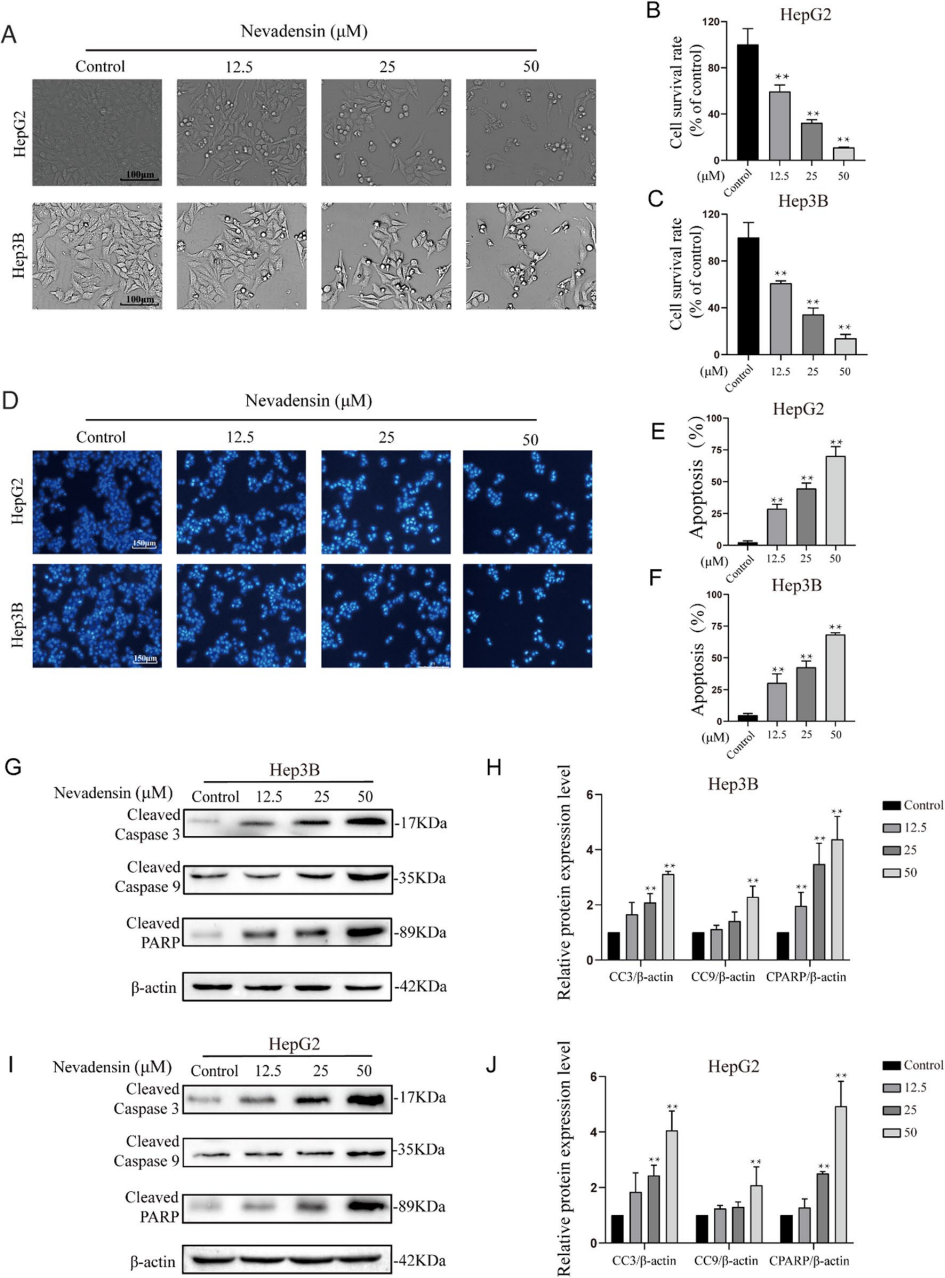
Nevadensin inhibits viability and induces apoptosis of HCC cells. **A** Hep3B and HepG2 cells were treated with different concentrations of nevadensin (ctrl, 12.5, 25, 50 µM) for 24 h. **B**, **C** Cell survival rate was plotted. n = 3, **p < 0.01, compared to control group. **D** Cells were treated with different doses of nevadensin for 24 h, followed by Hoechst33258 staining analysis. Representative images are shown. Blue highlights indicate apoptotic cells. **E**, **F** The apoptotic rate is plotted. n = 3, **p < 0.01, compared to control group. **G**–**J** The levels of Cleaved-PARP, Cleaved Caspase 3 and Cleaved Caspase 9 in nevadensin-treated Hep3B and HepG2 cells were examined by Western blotting. Quantitative analysis of the levels of Cleaved-PARP, Cleaved Caspase 3 and Cleaved Caspase 9 in nevadensin-treated Hep3B and HepG2 cells. n = 3, **p < 0.01, compared to control group

To determine the cytotoxicity of nevadensin on HCC cells, the Hep3B and HepG2 cells were incubated with different concentrations of nevadensin (ctrl, 12.5, 25, 50 µM) for 24 h. The results of this treatment showed that there was a significant increase in the number of floating and morphologically-shrunken cells when the concentration of nevadensin was increased (Fig. 1A). The cell survival rate (%) was estimated, and the results indicated that nevadensin could significantly attenuate the viability of Hep3B and HepG2 cells in a concentration-dependent manner (Fig. 1B, C).

Hoechst 33258 staining was used to determine whether apoptosis was responsible for attenuating the viability of the nevadensin-treated cells (Fig. 1D). The findings showed that nevadensin treatment significantly increased apoptosis compared to the control cells (Fig. 1E, F). Thereafter, the nevadensin-treated Hep3B (Fig. 1G, H) and HepG2 (Fig. 1I, J) cells were analyzed by means of Western blot analysis, and the results revealed that there was a concentration-dependent increase in the levels of a few apoptosis-related proteins, such as Cleaved Caspase 3, Cleaved-PARP, and Cleaved Caspase 9. Thus, it was concluded that apoptosis may be one of the factors that nevadensin inhibited the growth of the HCC cells.

### Nevadensin causes cell cycle arrest and attenuates the proliferation of HCC cells

**Fig. 2:**
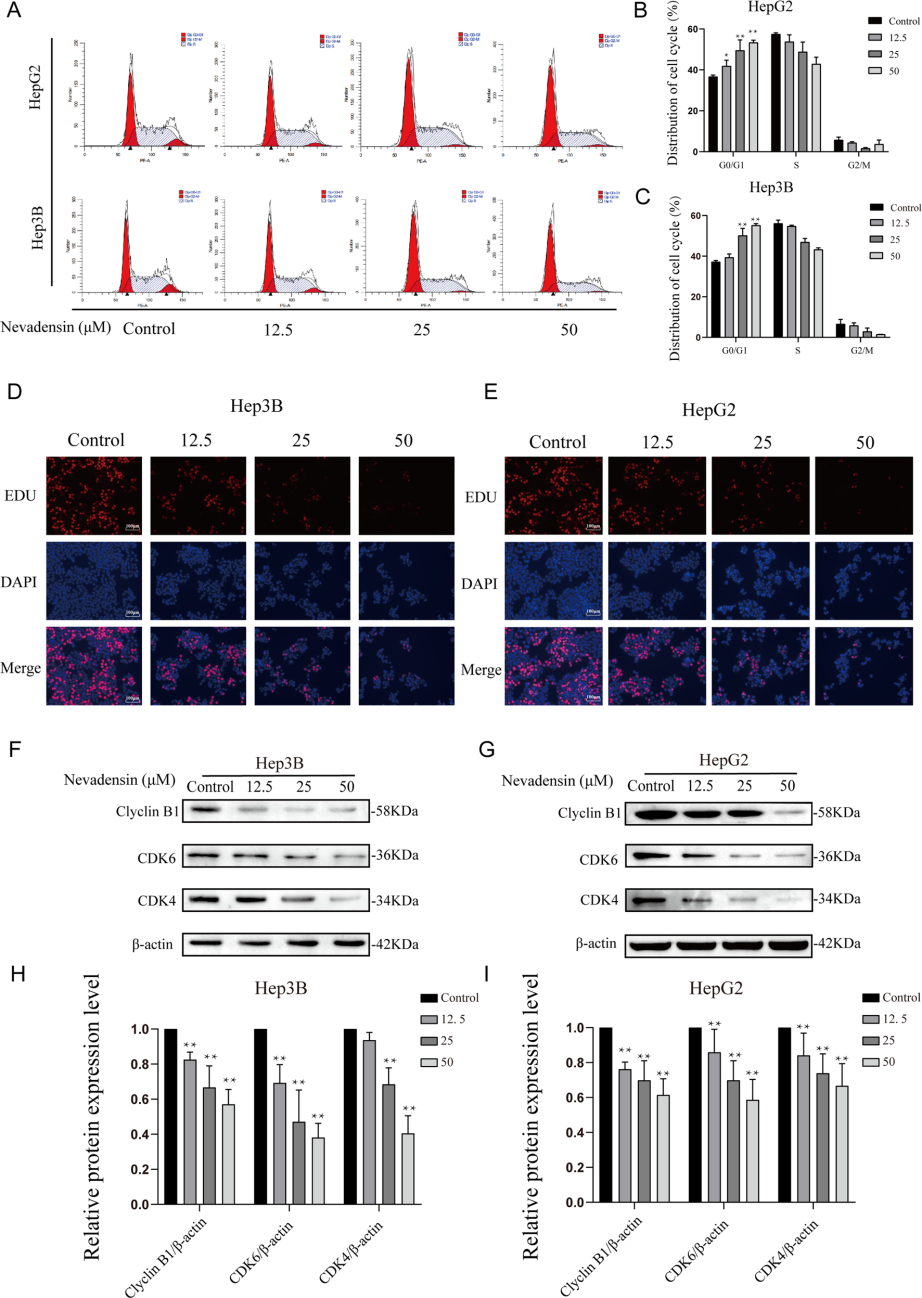
Nevadensin induces cell cycle arrest and reduces cell proliferation of HCC cells. **A** Cell cycle distributions of the Hep3B and HepG2 cells were measured by flow cytometry after treatment with various concentrations of nevadensin for 24 h. **B**, **C** Statistical analysis of the cell cycle distribution of nevadensin-treated the Hep3B and HepG2 cells. n = 3, * p < 0.05; ** p < 0.01 compared to control group. **D**, **E** Hep3B and HepG2 cells were treated with different doses of nevadensin for 24 h, followed by EdU labeling analysis. Representative images are shown. EdU-labeled (Red) indicates the proliferation of cells, DAPI (Blue) labeled nucleus, and Merge indicates combined immunofluorescence signals for EdU (Red) and DAPI (Blue). **F**, **G** The levels of Cyclin1, CDK4 and CDK6 in nevadensin-treated Hep3B and HepG2 cells were examined by Western blotting. **H**, **I** Quantitative analysis of the levels of Cyclin1, CDK4 and CDK6 in nevadensin-treated Hep3B and HepG2 cells. n = 3, **p < 0.01, compared to control group

It has been established that cell cycle arrest plays a significant role in inhibiting cell proliferation. A flow cytometric analysis was carried out to determine whether cell cycle arrest in HCC cells was related to nevadensin treatment, and the results indicated that the cell cycle in the nevadensin-treated HepG2 and Hep3B cells was arrested at the G0/G1 phase (Fig. 2A–C). The above findings were further validated by the EDU staining experiments. Furthermore, nevadensin treatment significantly reduced the growth of HepG2 and Hep3B cells (Fig. 2D, E). Western blotting analysis revealed that Cyclin 1, CDK4, and CDK6 levels were also significantly down-regulated following nevadensin treatment (Fig. 2F–I). These results suggested that inhibition in HCC cell growth could also be attributed to nevadensin-induced cell cycle arrest.

### Nevadensin regulates multiple molecular pathways that are involved in the hippo pathway activation in HCC cells

**Fig. 3:**
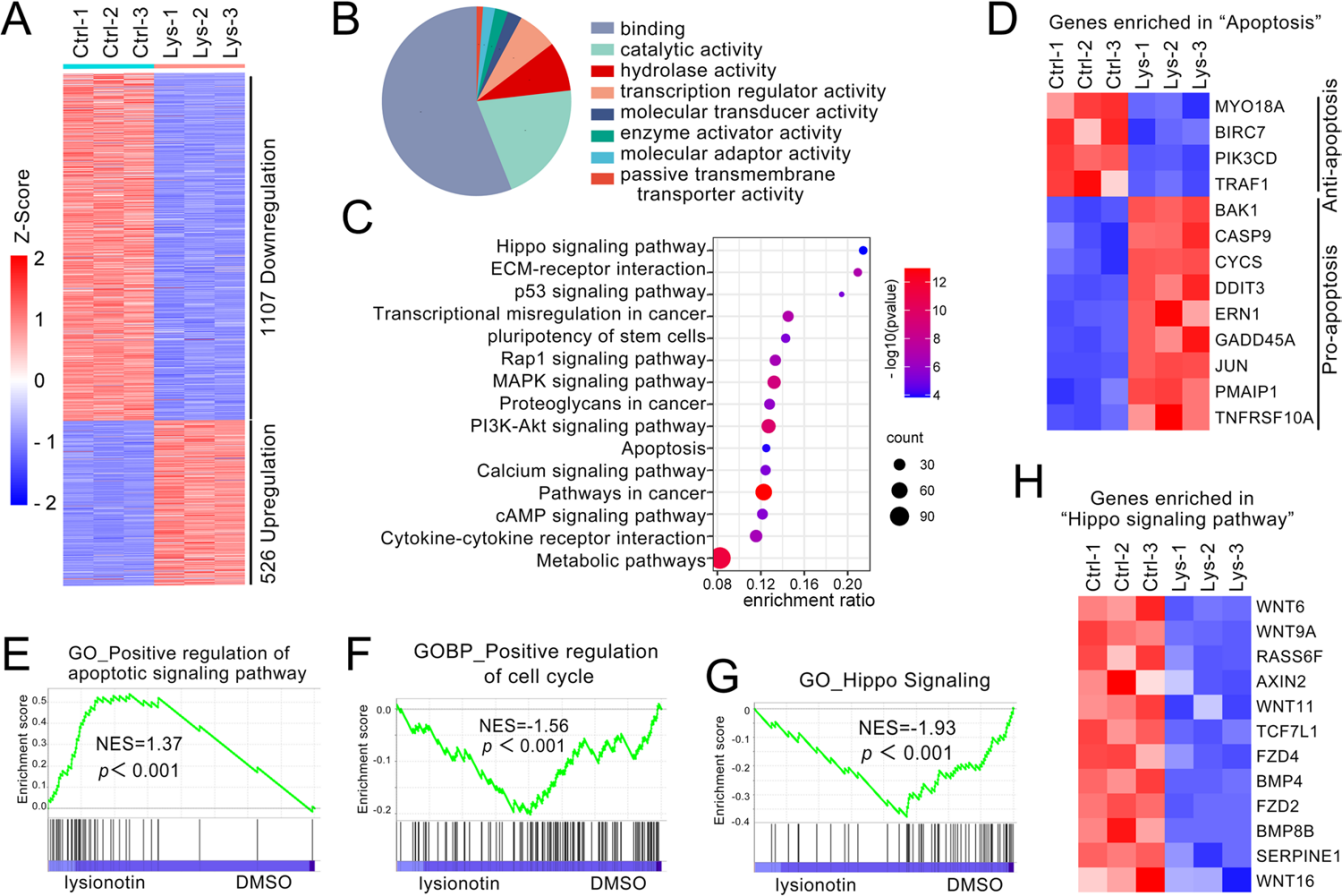
Nevadensin regulates multiple molecular pathways involved hippo pathway activation in HCC cells. **A** The gene expression profiles were assessed using RNAseq in nevadensin-treated Hep3B cells. **B** The differentially expressed genes (DEGs) were classified into eight functional groups. **C**, **D** A set of pre-defined genes were used for gene aggregation enrichment analysis of apoptosis signaling pathway and cell cycle in cells treated with different concentrations of nevadensin. **E** KEGG pathway analyses of the DEGs. **F** Analysis of genes enriched in the apoptosis signaling pathway. **G** A set of pre-defined genes were used for gene aggregation enrichment analysis of Hippo signaling pathway. **H** Analysis of genes enriched in the Hippo signaling pathway

Gene expression profiles were evaluated using RNAseq of Hep3B cells to understand the underlying molecular mechanisms via which nevadensin inhibited HCC cell proliferation. This technique analyzed 1633 differentially expressed genes. In total, 1107 of these genes were down-regulated, whereas 526 genes were observed to be up-regulated (Fig. 3A). The differentially expressed genes were categorized into eight functional groups, namely, binding, catalytic activity, transcription regulator activity, hydrolase activity, molecular transducer activity, molecular adaptor activity, enzyme activator activity, and passive transmembrane transporter activity (Fig. 3B), demonstrating that numerous cellular and molecular processes were altered in the nevadensin-treated cells. Gene set enrichment analysis (GSEA)-based annotation of the ribosomal-load mRNAs indicated an intriguing and unexpected increase in the number of genes that encoded for proteins involved in the positive regulation of the cell cycle (Fig. 3C) and the positive control of apoptotic signaling pathway (Fig. 3D). A majority of these KEGG pathways were divided into two categories: Group 1 including the up-regulated pro-apoptosis pathways, which were one of the most important pathways; and Group 2 including the down-regulated anti-apoptosis pathways (Fig. 3F). To assess the nevadensin-activated signaling pathways, KEGG pathway analysis of the above differentially expressed genes revealed the enrichment of the Hippo signaling system, the pluripotency of stem cells, p53 signaling pathway, ECM-receptor interaction, transcriptional misregulation in cancer, MAPK signaling pathway, Rap1 signaling pathway, proteoglycans in cancer, Apoptosis, PI3K-Akt signaling pathway, Calcium-signaling pathways, Cytokine-cytokine receptor interaction, cAMP signaling pathway, and Metabolic pathways (Fig. 3E). Subsequently, GSEA was conducted to determine if the nevadensin-treated cells showed a differential expression of the pre-defined set of genes in contrast with the control samples. Consistent with the results of Fig. 3E, GSEA analysis indicated that the misregulated genes showed enrichment for the gene set that was linked to the Hippo signaling pathway (Fig. 3G) and other genes that were enriched in “Hippo signaling pathway”, such as “WNT6, WNT9A, RASS6F, AXIN2, WNT11, TCF7L1, FZD4, BMP4, FZD2, BMP8B, SERPINE, and WNT16” (Fig. 3H). The results indicated that these genes were down-regulated in the nevadensin-treated cells. The findings also showed that the activation of the Hippo signaling pathway could be responsible for the nevadensin-induced cell apoptosis and arrested cell cycle.

### Nevadensin induces Hippo-ON ***via*** MST1/2- LATS1/2-YAP signaling activation

**Fig. 4:**
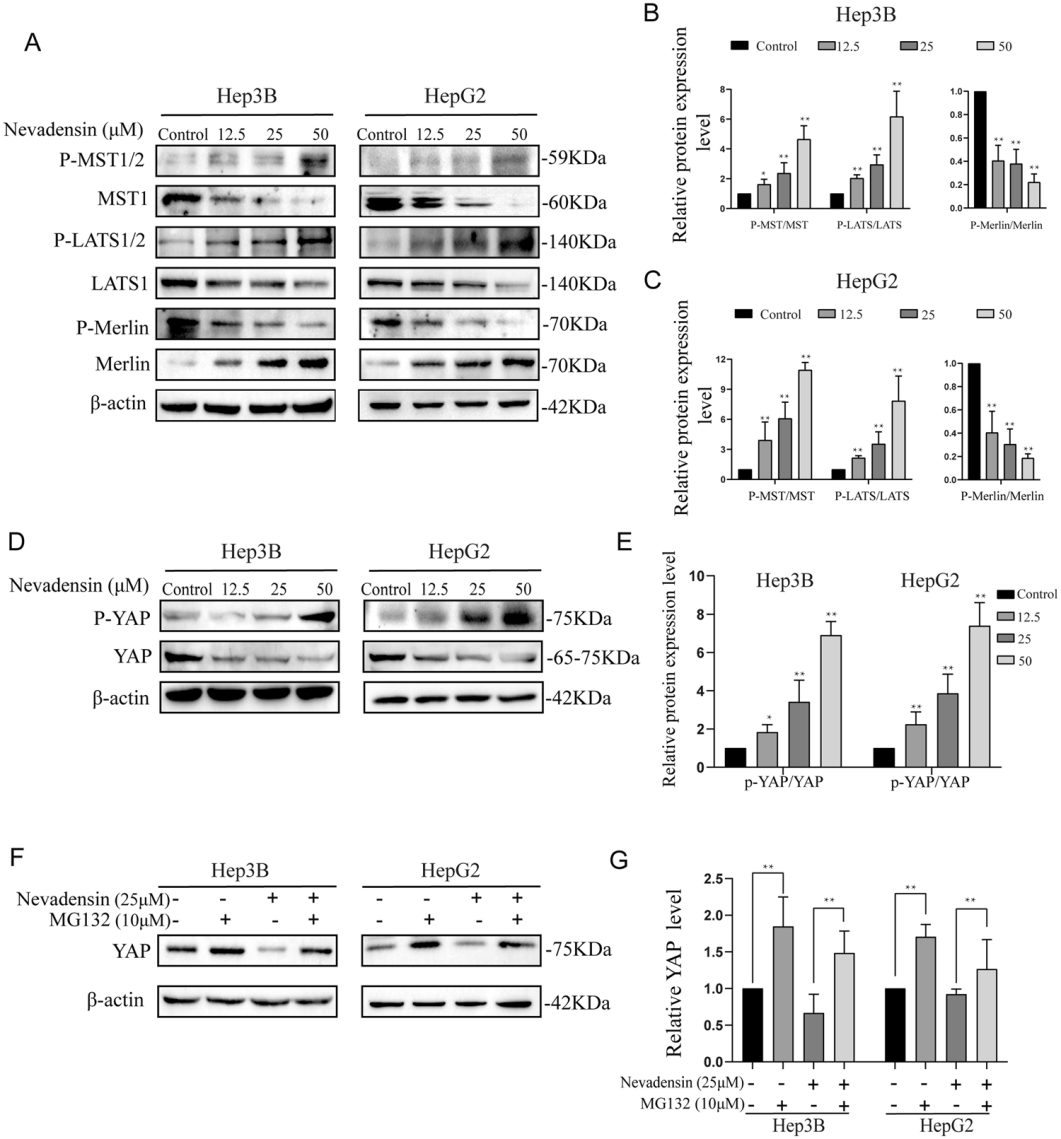
Nevadensin induces Hippo-ON via MST1/2- LATS1/2-YAP signaling activation. **A** The levels of Phospho-MST, MST, Phospho-LATS, LATS, Phospho-Merlin and Merlin in nevadensin-treated Hep3B and HepG2 cells were examined by Western blotting. **B**, **C** Quantitative analysis of the levels of Phospho-MST/MST, Phospho-LATS/LATS, Phospho-Merlin /Merlin in nevadensin-treated Hep3B and HepG2 cells. n = 3, *p < 0.05; **p < 0.01, compared to control group. **D** The levels of Phospho-Yap and yap in nevadensin-treated Hep3B and HepG2 cells were examined by Western blotting. **E** Quantitative analysis of the levels of Phospho-Yap/Yap in nevadensin-treated Hep3B and HepG2 cells. n = 3, *p < 0.05; **p < 0.01, compared to control group. **F** The levels of Yap in Hep3B and HepG2 cells after treatment with nevadensin (12.5 µM), MG132 (10 µM) or both for 24 h were examined by Western blotting. **G** Quantitative analysis of the levels of Yap in treated Hep3B and HepG2 cells. n = 3, *p < 0.05; **p < 0.01, compared to control group

Both the results from KEGG and GSEA analysis promote us to verify the effect of nevadensin on the Hippo pathway. Here, the expression of various Hippo pathway-related factors in the nevadensin-treated HCC cells was examined. Western blot analysis indicated that the MST1/2 phosphorylation and LATS1/2 phosphorylation levels were up-regulated by nevadensin, however, the whole MST1/2 and LATS1/2 expression levels were decreased in the HepG2 and Hep3B cells (Fig. 4A–C). These suggested that nevadensin could activate the Hippo-On mechanism via phosphorylation, thereby activating the MST1/2-LATS1/2 pathway (Fig. 4A–C). Another essential protein molecule, called “Merlin”, was also identified in the hippo pathway. Since Merlin is a well-studied tumor suppressor, the whole Merlin-upregulation process could activate Hippo-ON. As anticipated, nevadensin treatment in the HepG2 and Hep3B cells significantly enhanced the complete Merlin assembly and down-regulated the phosphorylated Merlin molecule (Fig. 4A–C).

Previous studies have shown that Yap was involved in the tumorigenesis and development of malignant cells [28]. It is especially involved in the Hippo pathway since it acts as a major effector molecule that can regulate cell proliferation. Hence, experiments were conducted to determine whether nevadensin regulated Yap through the Hippo pathway, which, in turn, affected cell proliferation. Western blot showed that Yap expression was downregulated in a dose-dependent manner, and the phosphorylation level of Yap was elevated (Fig. 4D–F). HepG2 and Hep3B cells were treated with ctrl or nevadensin, followed by treatment with or without MG132, a proteasome inhibitor, to confirm the effects of nevadensin on Yap expression. MG132 treatment prevented the down-regulation of Yap caused by nevadensin treatment, whereas nevadensin treatment resulted in a decrease in Yap expression levels (Fig. 4F, G). The data suggested that nevadensin-triggered down-regulation of Yap could mainly induce protein degradation. The data further showed that nevadensin could induce Yap degradation by phosphorylating and activating the MST1/2-LATS1/2 pathway and Merlin, thus affecting the proliferation of tumor cells.

### Down-regulation of YAP by nevadensin can sensitize HCC cells to sorafenib

**Fig. 5:**
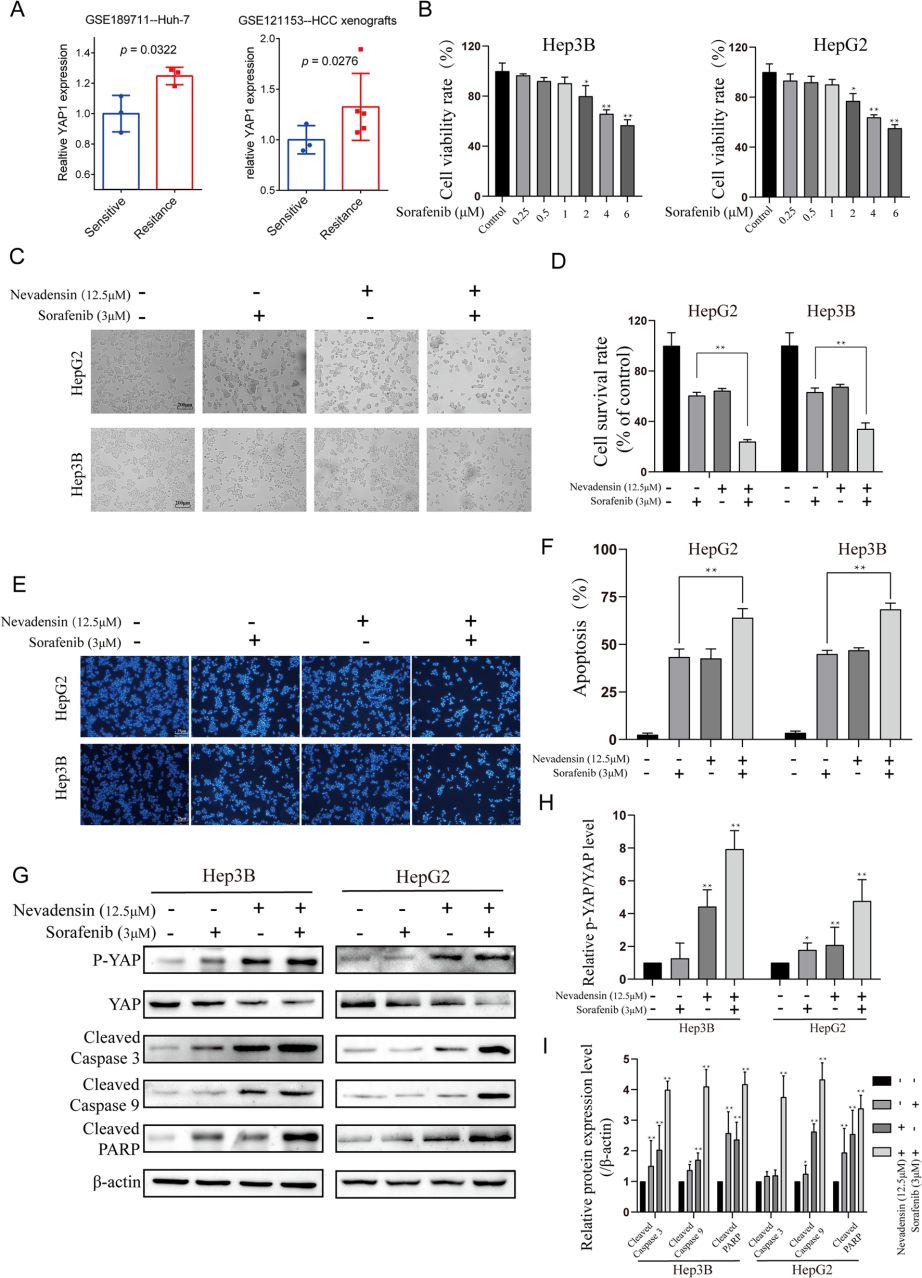
Down-regulation of YAP by nevadensin sensitizes HCC cells to sorafenib. **A** YAP in hepatocarcinoma cell lines Huh7 (GSE189711, p = 0.0322) and HCC xenografts (GSE121153, p = 0.0276) resistant to sorafenib was analyzed using database. **B** After treatment with different concentrations of sorafenib (ctrl, 0.25, 0.5, 1, 2, 4, 6 µM) for 24 h, the cell viabilities of Hep3B and HepG2 cells were determined using the cell counting kit-8 assay. The cell viabilities rate is plotted. n = 3, **p < 0.05; **p < 0.01, compared to control group. **C**, **D** HepG2 and Hep3B cells were treated with nevadensin (12.5 µM), sorafenib (3 µM) or both for 24 h. Cell survival rate was plotted. n = 3, **p < 0.01, compared to control group. **E**, **F** Hep3B and HepG2 cells were treated with nevadensin (12.5 µM), sorafenib (3 µM) or both for 24 h, followed by Hoechst33258 staining analysis. Representative images are shown. Blue highlights indicate apoptotic cells. The apoptotic rate is plotted. n = 3, **p < 0.01, compared to control group. **G** The levels of Phospho-Yap, Yap, Cleaved-PARP, Cleaved Caspase 3 and Cleaved caspase 9 in Hep3B and HepG2 cells treated with different drugs were examined by Western blotting. **H** Quantitative analysis of the levels of Phospho-Yap/Yap in treated Hep3B and HepG2 cells. n = 3, *p < 0.05; **p < 0.01, compared to control group. **I** Quantitative analysis of the levels of Cleaved-PARP, Cleaved Caspase 3 and Cleaved Caspase 9 in treated Hep3B and HepG2 cells. n = 3, *p < 0.05; **p < 0.01, compared to control group

Although sorafenib is a first-line therapeutic drug used for advanced HCC, subsequent drug resistance makes it less effective. Database analysis found that the high expression of YAP in hepatoma cell lines such as Huh7 (GSE189711, p = 0.0322) and HCC xenografts (GSE121153, p = 0.0276) conferred more resistance to sorafenib (Fig. 5A). These results suggested that high YAP expression might be one of the causes of sorafenib resistance. The above results clearly showed that nevadensin could inhibit the YAP activity, and improve the sorafenib resistance to Yap.

**Fig. 6:**
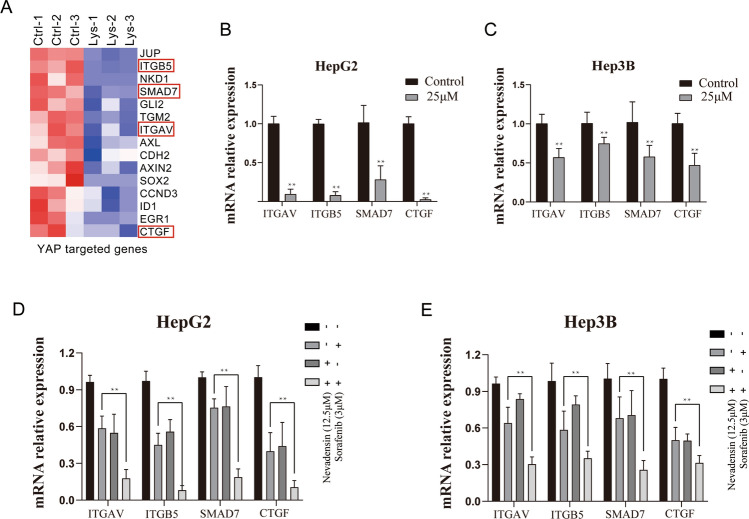
Nevadensin sensitizes HCC cells to sorafenib via downregulating expression of YAP target genes. **A** Genes with decreased expression of YAP target genes in Hep3B cells after administration. **B**, **C** HCC cells were treated with 25 µM nevadensin for 24 h; **D**, **E** HCC cells were treated with nevadensin (12.5 µM), sorafenib (3 µM) or both for 24 h; followed by RT-PCR analysis of transcripts of YAP target genes (ITGB5, SAMD7, ITGAV and CTGF). These mRNA levels were normalized to GAPDH. The values represent the mean ± standard deviation. n = 3, **p < 0.01, compared to control

Initially, CCK-8 experiments were conducted to screen the sorafenib concentration (Fig. 5B). Then, HepG2 and Hep3B cells were treated with nevadensin, sorafenib, or both, followed by tests to determine cell proliferation and apoptosis. The results showed that the drug combination group showed a significant decrease in proliferation (Fig. 5C, D) and increase in apoptosis (Fig. 5E, F), compared with nevadensin or sorafenib alone. Although treatment with nevadensin or sorafenib alone showed little effect on the Yap expression and Yap activity, the combination of nevadensin and sorafenib drugs showed lower YAP levels compared to cells that were treated with sorafenib or sorafenib alone (Fig. 5G–I). For combination experiments, 12.5 µM of nevadensin was used, which was not effective when used alone, however, it improved the effect of sorafenib. Also, the combination of nevadensin and sorafenib treatments in the cells could significantly elevate the level of apoptotic proteins (cleaved Caspase 9, cleaved Caspase 3, cleaved PARP) compared to only nevadensin- or sorafenib-treated cells (Fig. 5G–I). Furthermore, several common downstream effectors of Yap were selected for PCR validation, and the results showed that many Yap targets (such as ITGB5, SAMD7, ITGAV, and CTGF) showed a lower expression compared with nevadensin treatment (Fig. 6A–C). Consistent with these results, the combination of nevadensin and sorafenib treatments in the cells could significantly lower the transcriptional level (like ITGB5, SAMD7, ITGAV, and CTGF) compared to only nevadensin- or sorafenib-treated cells (Fig. 6D, E). In conclusion, these results suggested that Yap-induced insensitivity of HCC to sorafenib could be reversed by nevadensin.

## Discussion

HCC shows the fastest-rising incidence rate among all cancers and is reported to be the leading cause of cancer-related deaths, globally, particularly in China. Studies conducted over the past ten years have revealed a strong correlation between the dysregulation of the Hippo pathway and a variety of human malignancies. Any changes occurring in the major proteins associated with the Hippo pathway results in malignant cell invasion, disease progression, cell migration, and treatment resistance. Multiple efforts have been made to target key components of this pathway for various types of cancer intervention. In this work, nevadensin, which inhibits YAP, may be used as a strategy to increase the effectiveness of sorafenib in treating HCC and to identify a few novel molecular events that occur in HCC cells following nevadensin treatment. Here, cleaved caspase-3 and PARP levels were increased in nevadensin-treated HCC cells, which also led to cell cycle arrest by down-regulating cyclin B, cyclin D, CDK4, and other proteins. The major elements of the Hippo pathway, such as activating the MAST1/2-LATS1/2 phosphorylation, activating the merlin phosphorylation, as well as Yap phosphorylation and degradation, are involved in the suppression of numerous Yap targeted genes, which is consistent with the results of RNAseq. Furthermore, high Yap expression is often accompanied by sorafenib resistance. The results also indicated that nevadensin sensitized the HCC cells to sorafenib treatment by inhibiting Yap (Fig. 6). These findings suggested that the combination of nevadensin and sorafenib could be used as a novel strategy for the treatment of liver cancer.

In this study, the results showed that nevadensin suppressed cell cycle and triggered apoptosis. However, the exact molecular mechanism is yet unknown. The RNAseq data showed that nevadensin activated the hippo pathway. The traditional hippo-Yap-pathway-kinase-cascade indicated that the MST1/2-SAV1 complex phosphorylated and activated the LATS1/2-MOB1A/B molecules, followed by the phosphorylation and inactivation of YAP/TAZ. MST1/2 and LATS1/2 are the major kinases of the cascade, whereas SAV1 and MOB1A/B serve as adaptor proteins to increase the phosphorylation and activation of the above kinases [19]. Additionally, nevadensin could activate the key Hippo pathway proteins like MST1/2 and LAST1/2 while inactivating Merlin. Merlin, also known as the protein neurofibromin 2 (NF2), is a widely studied tumor suppressor that activates the Hippo pathway to inhibit YAP/TAZ activity via the mechanism of targeting LATS1/2 and improving the phosphorylation and activation of YAP/TAZ by MST1/2 [29]. Additionally, localization of the YAP/TAZ gene within the cells and its protein level are frequently used as indicators of the hippo pathway activation. The nevadensin-treated HCC cells significantly phosphorylated and activated MST1/2-LATS1/2, phosphorylated and inactivated Merlin, and enhanced the activity of the Hippo pathway to inhibit the YAP/TAZ activity, eventually inhibiting the growth of HCC cells.

Additionally, Yap, a core protein of the hippo pathway, is overexpressed in human HCC tumors and is vital for the proliferation of HCC cells. Abrogating activated Yap in the existing hepatoblastoma also results in in vivo tumor regression [30]. The data showed that Yap was phosphorylated and then degraded in the nevadensin-treated HCC cells, which was consistent with earlier findings. The target genes of YAP/TAZ included those that regulated a variety of cell phenotypes, such as cell proliferation, migration, extracellular matrix organization, epithelial-mesenchymal transition, cytoskeletal organization, etc. [31–33]. According to the KEGG and GO analysis results, nevadensin treatment also suppressed the Yap-targeted genes, including ITGB5, SMAD7, ITGAV, and CTGF. When the hippo signaling pathway is OFF, YAP/TAZ penetrates the nucleus and engages with the TEF/TEAD family of DNA-binding transcription factors, which includes TEAD1/2/3/4, to promote the Hippo target genes, such as CTGF [34]. Consistent with the results observed in this study, the PCR results also demonstrated that the YAP target genes were suppressed during the transcription process, in the nevadensin-treated cells.

An earlier study showed that the YAP/TAZ and Hippo pathways could be involved in regulating the WNT pathways in specific cell types and environments [35, 36]. In this study, it was noted that multiple WNTs genes involved in controlling the hippo pathway were downregulated after nevadensin treatment, and were enriched in the hippo signaling pathway. Additionally, YAP overexpression in the hepatocytes could promote HCC onset and progression [37]. In particular, it was noted that the depletion of Mst1/2 [38–40] could cause HCC, which highlighted the significance of the Hippo pathway in restricting the growth of liver cancer. Additionally, in an earlier study, the researchers developed a YAP/TAZ gene signature that identified the patients with unfavorable prognoses [41]. Numerous studies showed that higher YAP and TAZ levels were associated with a worse prognosis in liver cancer patients [42–44], and a majority of HCC patients (62%) showed YAP overexpression. Previous research has demonstrated that YAP plays a role in the development of cancer resistance [45, 46]. Consequently, YAP is regarded as a promising therapeutic target for cancer. Recent research revealed that the Hippo pathway was closely linked to a poor prognosis and cancer cells’ responses to chemotherapeutic treatments [41–44]. Therefore, it is crucial to find promising strategies for overcoming Hippo-induced chemotherapy resistance. For example, both previous and recent studies have demonstrated that YAP/TAZ, as the major component of the Hippo pathway, was responsible for chemotherapeutic resistance in HCC, such as cisplatin (CDDP) and doxorubicin (Dox) [45–49]. Moreover, Sun et al. found that sorafenib enhanced the accumulation and activation of nucleus YAP, thereby promoting the resistance of sorafenib by inhibiting apoptosis of HCC cells through the downstream mediator of YAP and survivin [50]. The effectiveness of the medications that target YAP and lead to resistance in HCC may also be improved by inhibiting YAP.

Sorafenib, in particular, targets cancer cells by disrupting intracellular protein kinase cascades and is used as the standard treatment for advanced HCC [51]. However, the lack of effective alternative systemic therapy options raises serious concerns about sorafenib-related resistance [52]. In their study, Gao et al. integrated the genome-wide synthetic lethality screen with transcriptome analysis, to identify the molecular causes of sorafenib resistance. These combined techniques identified Yap/Taz (popular Hippo signaling pathway transducers) as important mediators of sorafenib resistance [50, 53–56]. Previous research suggested that Yap inhibition would be feasible in clinical settings. A viable therapeutic approach for advanced HCC may also involve combining Yap inhibitors with sorafenib [50]. A photosensitizer called verteporfin is clinically used to treat macular degeneration. It is the only small molecule capable of directly targeting the transcriptional activity mediated by YAP-TEAD. It has been discovered that verteporfin has shown effectiveness in inhibiting HCC cell growth both in vitro and in vivo [57]. Using YAP RNAi- lipid nanoparticles (siYAP-LNP) are another way to inhibit YAP. Fitamant et al. demonstrated that siYAP-LNP could effectively induce HCC regression in liver-specific Mst1/Mst2 knockout mice [58]. The results of our studies showed that nevadensin could improve the efficacy of sorafenib resistant to Yap, it suggested that nevadensin might be a promising inhibitor of Yap. Although, extensive investigation using orthotopic xenograft models is required to confirm if nevadensin decreases the in vivo expression of YAP. These results could offer insights into the development of novel medications, like nevadensin analogs that could overcome sorafenib resistance in HCC by targeting the hippo-Yap pathway.

Additionally, there are certain limitations to this study. The anti-HCC effects of nevadensin were only evaluated in two human hepatocellular carcinoma cell lines, HepG2 and Hep3B, and thus other liver cancer cells will be using to further explore the therapeutic effect of nevadensin in subsequent studies. Furthermore, the effects of nevadensin in a HepG2-xenografted model in nude mice warrant further validation. In addition, although this study revealed that nevadensin could induce the Hippo-ON mechanism by activating the MST1/2- LATS1/2 kinase in HCC cells, further studies are needed to determine which RNF molecules are involved in the regulation of MST1/2 by nevadensin. Moreover, novel molecular targets of nevadensin deserve to be identified in HCC as well as other types of cancers.

## Conclusions

**Fig. 7:**
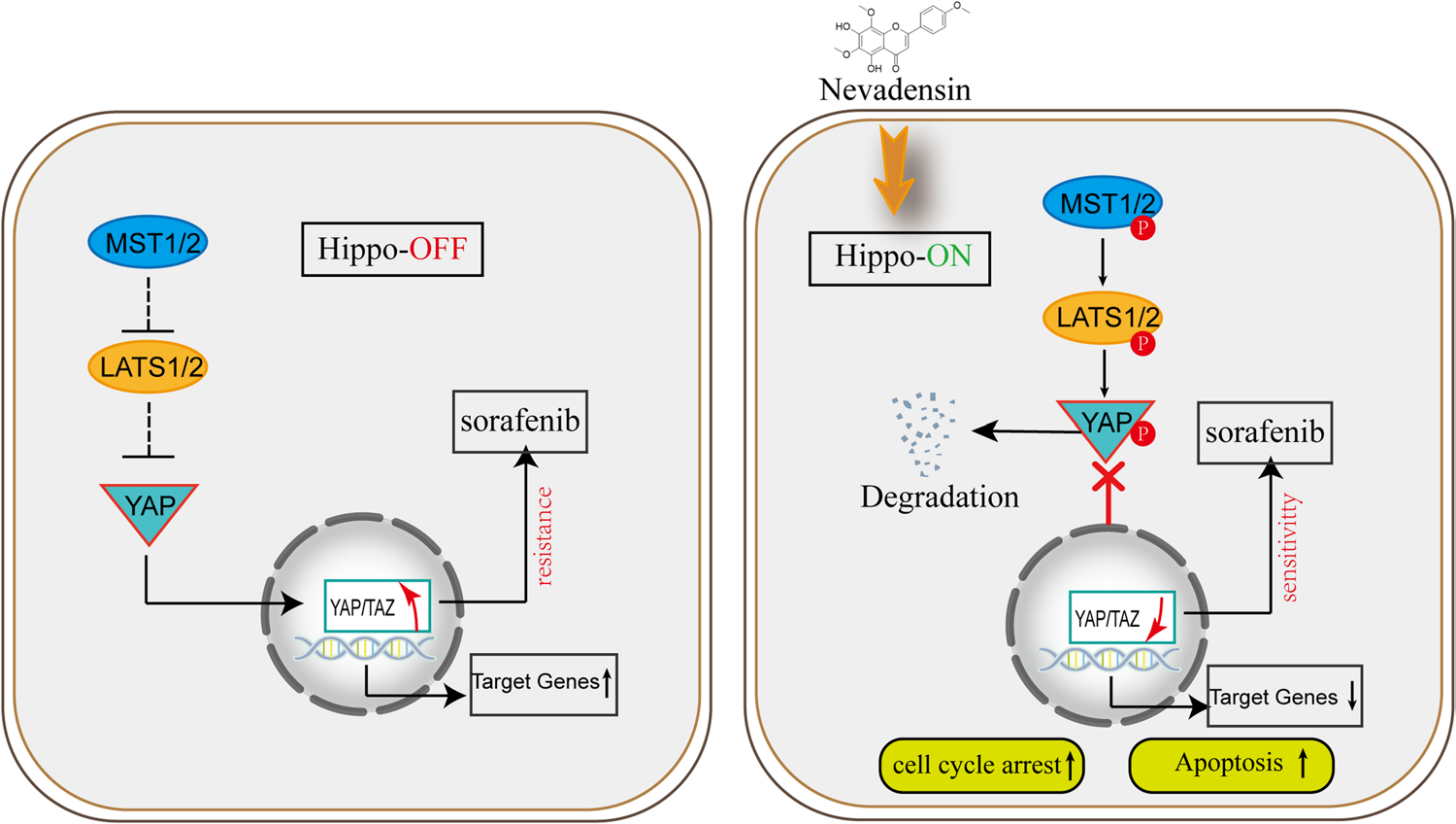
Proposed working model of how Nevadensin regulates viability of HCC cells

This study observed that nevadensin was involved in the activation of the hippo-Yap pathway during the development of sorafenib resistance in HCC. Herein, we reported that Hippo-off promotes activation of YAP/TAZ subsequently induces cancer cell growth and sorafenib resistance in liver cancer. Conversely, nevadensin notably induces Hippo-on via activating the MST1/2-LATS1/2 kinase, further resulting in phosphorylation and degradation of YAP. Therefore, nevadensin remarkbaly induces cell cycle arrest and apoptosis, and synergistically sensitizes liver cancer cells to sorafenib (Fig. 7). These results indicated that nevadensin might exert its anti-HCC activity through the Hippo-ON mechanism. Moreover, nevadensin could increase the sensitivity of HCC cells to sorafenib by down-regulating YAP and its downstream targets. This study raises the possibility and offers the first proof of concept that targeting Yap could be used as an effective strategy to overcome therapy resistance. Finally, this study presents the first pre-clinical evidence that the precise targeting of Yap implicated in its targeting gene may present alluring chances to overcome sorafenib and possibly other therapeutic resistance since sorafenib resistance acts as a key clinical hurdle in the treatment of HCC.

## Data Availability

The datasets generated during the current study are available from the corresponding author on reasonable request.
